# On the Leading Edge of Longevity: Lessons From the MIT AgeLab 85+ Lifestyle Leaders

**DOI:** 10.1093/geroni/igaa057.2070

**Published:** 2020-12-16

**Authors:** Julie Miller, Lisa D’Ambrosio

## Abstract

The 85+ population is the fastest-growing age segment in the United States. Understanding attitudinal and behavioral trends among adults ages 85 and over is of increasing importance to researchers and practitioners alike. This symposium will present findings drawing on mixed methods across multiple domains of research with the Lifestyle Leaders, a bimonthly panel study of adults ages 85 and older at the MIT AgeLab, which began in September 2015. Each presentation in this symposium will highlight a different focus of research conducted with the Lifestyle Leaders, ultimately offering insights about physical health, social engagement, and civic engagement at age 85 and beyond. The first presentation in this symposium will frame Lifestyle Leaders’ resources and how these resources affect their perceptions of risks, their worries, and their well-being. Diving deeper, the second presentation will center on how Lifestyle Leaders perceive and utilize different transportation options and how these options have changed for them over time. The third presentation will focus on civic engagement and Lifestyle Leaders’ perspectives on technology as enabling or inhibiting their civic engagement and participation. The fourth presentation will examine Lifestyle Leaders’ attitudes toward medication, medication management, and technology-enabled medication management devices. This symposium will deepen attendees’ understandings of the attitudes and experiences of active octogenarians and nonagenarians.

